# Effectiveness of immunoglobulin replacement therapy in preventing infections in patients with chronic obstructive pulmonary disease: a systematic review

**DOI:** 10.1186/s13223-024-00886-8

**Published:** 2024-04-10

**Authors:** Justin J. Y. Kim, Liz Dennett, Maria B. Ospina, Anne Hicks, Harissios Vliagoftis, Adil Adatia

**Affiliations:** 1 Faculty of Medicine, University of Alberta, Edmonton, AB Canada; 2 Department of Medicine, University of Alberta, Edmonton, AB Canada; 3 Sperber Health Sciences Library University of Alberta, Edmonton, AB Canada; 4 Department of Public Health Sciences, Queen’s University, Kingston, ON Canada; 5 Department of Pediatrics, University of Alberta, Edmonton, AB Canada; 6 Alberta Respiratory Centre, University of Alberta, Edmonton, AB Canada

**Keywords:** Chronic obstructive pulmonary disease, Hypogammaglobulinemia, Immunoglobulin therapy, Primary immunodeficiency, Inborn errors of immunity, Systematic review

## Abstract

**Purpose:**

Immunoglobulin replacement therapy is a standard treatment for patients with antibody production deficiencies, which is of interest in patients with chronic obstructive pulmonary disease (COPD). This systematic review, registered with PROSPERO (CRD42021281118), assessed the current literature regarding immunoglobulin replacement therapy on COPD clinical outcomes in patients with low immunoglobulin G (IgG) serum concentrations.

**Methods:**

Literature searches conducted from inception to August 23, 2021, in databases including MEDLINE, EMBASE, and CINAHL. Population (sex, age, comorbidities), baseline clinical characteristics (pulmonary function testing results, IgG levels), and outcome (hospitalizations, emergency department visits) were extracted after title/abstract and full text screening. The Cochrane risk of bias assessment form was used for risk of bias assessment of randomized controlled trials and the National Heart, Lung, and Blood Institute (NHLBI) assessment was used for pre and post studies.

**Results:**

A total of 1381 studies were identified in the preliminary search, and 874 records were screened after duplicates were removed. Screening 77 full texts yielded four studies that were included in the review.

**Conclusion:**

It is unclear whether immune globulin replacement therapy reduces acute exacerbation frequency and severity in COPD. Current evidence suggests that it is worth considering, but better developed protocols for administration of immune globulin supplementation is required for future randomized controlled trials.

**Supplementary Information:**

The online version contains supplementary material available at 10.1186/s13223-024-00886-8.

## Introduction

Chronic obstructive pulmonary disease (COPD) is a respiratory condition characterized by airflow limitation, and it is one of the leading causes of death worldwide [1, 2]. Approximately 212.3 million people globally were reported to have COPD in 2019, and the prevalence is increasing [3]. COPD is marked by progressive chronic symptoms and episodic acute exacerbations (AECOPD) that further impair quality of life, worsen lung function [4], and increase the risk of mortality [5].

The most common aetiologies of AECOPD are respiratory tract infections followed by eosinophilic airway inflammation, and both occur simultaneously in some patients [6–9]. The most frequently implicated pathogens in AECOPD are bacteria including *Haemophilus influenzae, Streptococcus pneumoniae,* and *Moraexlla catarrhalis,* and respiratory viruses including influenza and parainfluenza viruses, rhinoviruses, and coronaviruses. Strategies to reduce the burden of respiratory infections are thus of significant interest to prevent AECOPD [10].

Studies have shown that approximately 25% of COPD patients have reduced immunoglobulin G (IgG) serum concentrations [11], and that this finding is independently associated with AECOPD and hospitalization risk [12–14]. IgG serum concentrations and AECOPD severity also appear to be inversely related [15, 16]. Given the well-established role of IgG in host defense against respiratory infections, these data have raised the question of whether treatment of low IgG in this patient population may reduce AECOPD risk [17].

Immune globulin is a plasma-derived therapeutic product consisting of IgG obtained from thousands of blood donors [18]. Immune globulin therapy (IGT) is a well-established treatment modality to prevent infections in patients with primary and secondary antibody deficiencies [19]. Both intravenous (IVIG) and subcutaneous (SCIG) treatment significantly reduce the frequency and severity of bacterial respiratory infections in this patient population [15, 20]. However, IGT is expensive and adverse reactions are common among those receiving IVIG [21]. Therefore, it is imperative that the effectiveness of IGT is well validated prior to its regular usage as adjunct therapy in COPD.

We conducted a systematic review to understand whether IgG replacement therapy for patients with COPD and decreased serum IgG levels decreases AECOPD frequency.

## Methods

The review was registered with PROSPERO (CRD42021281118) prior to initiation and reported according to the Preferred Reporting Items for Systematic Reviews and Meta-Analyses (PRISMA) guidelines [22].

### Search strategy

Comprehensive searches were completed in the following databases from inception to August 23, 2021, to identify relevant publications: MEDLINE, Embase (Ovid interface), CINAHL Plus with Full Text (EBSCOhost interface), Cochrane Library Trials database (Wiley Interface), Scopus, Web of Science Core Collection, and Google Scholar (on August 27, 2021). The search used a combination of subject headings and relevant keywords related to COPD and IGT and was conducted by a health sciences librarian (LD) experienced in systemic review methodology (as detailed in Table 1). The following grey literature formats were included: conference abstracts (from Embase and Web of Science), clinical trial registry records (from Cochrane Trials), and publications from the Google Scholar search. The reference lists of relevant articles were screened, and authors of the articles of interest were contacted to ask for any relevant raw data. No language or publication types were restricted during the search.

**Table 1:** Search strategy for systematic review

COPD and immunoglobulin therapy search
1. Obstructive airway disease/or airway obstruction/or bronchus obstruction/or chronic obstructive lung disease/
2. Chronic bronchitis/
3. (Obstruct* adj3 (pulmonary or lung* or airway* or airflow* or bronch* or respirat*)).mp
4. ((Chronic* adj3 bronchiti*) or emphysema* or refractory asthma or chronic obstructive asthma or COPD or COAD or COBD or AECB).mp
5. Exp lung emphysema/
6. 1 or 2 or 3 or 4 or 5
7. Immunoglobulin G/ad, cm, cr, dv, do, it, dt, im, ip, tl, tr, iv, po, cj, sc, tp, td, tm [Drug Administration, Drug Comparison, Drug Concentration, Drug Development, Drug Dose, Drug Interaction, Drug Therapy, Intramuscular Drug Administration, Intraperitoneal Drug Administration, Intrathecal Drug Administration, Intratracheal Drug Administration, Intravenous Drug Administration, Oral Drug Administration, Subconjunctival Drug Administration, Subcutaneous Drug Administration, Topical Drug Administration, Transdermal Drug Administration, Unexpected Outcome of Drug Treatment]
8. (((Immunoglobulin* or IGG or IG or immune globulin* or gamma globulin* or gammaglobulin*) adj6 (therap* or treatment* or sub?cutaneous or intravenous or intra-venous or IV or oral or administration)) or IVIG).mp
9. 7 or 8
10. 6 and 9
11. Limit 10 to (animals and animal studies)
12. 10 not 11

### Study selection

Two reviewers (JK and HV) independently screened the titles and abstracts retrieved from the searches for relevance. Studies were eligible for inclusion if the included COPD patient populations have documented decreased serum IgG levels (serum IgG levels < 7/gL). Studies that include immunoglobulin replacement therapy (IVIG or SCIG) in addition to usual medical treatment for COPD (i.e. bronchodilators, inhaled steroids, antibiotics, oral steroids) were included. Studies with no outcome measure of COPD severity and symptoms and patient populations with asthma-COPD overlap syndrome (ACOS) were excluded. The primary outcomes included AECOPD frequency, hospitalizations and emergency department (ED) visits for AECOPD, and mortality from AECOPD events. The screening process was completed in Covidence. The full texts of relevant studies were then independently reviewed, and study authors were contacted for additional information on patient populations and outcomes, as required. A consensus was reached by discussion for to resolve all conflicts, which was managed by Covidence (Additional file 1).

### Risk of bias

Two reviewers (JK and AA) independently assessed the risk of bias of all included studies. The Cochrane risk of bias assessment was used for randomized controlled trials (RCT) [23]. Risk of bias categories included low risk, high risk, or unclear risk of bias for participant random allocation, patient selection, and outcome assessment. For before and after studies, the risk of bias assessment tools from the NHLBI was used [24]. Sources of bias were graded, and the differences in bias assessment were settled through a discussion until consensus was reached.

### Data extraction and synthesis of results

Data was independently extracted by both reviewers (JK and HV), and the items to be reported were finalized by consensus. Data extraction tables were created from the extracted raw data in Microsoft Excel. The data tables include specific study characteristics (study design, enrollment dates for treatment, location, funding, conflicts of interest), patient demographics (sample size, method of recruitment, sex, mean age, smoking history, and comorbidities), intervention (IgG route of administration and dosage), and clinical baseline characteristics and outcomes (pulmonary function testing (PFT) results, Ig levels). Authors of two studies with incomplete or unclear data were contacted but did not respond. Studies with incomplete or unclear data were not included in the final data extraction.

Due to the heterogeneity of the population characteristics, comparisons made, and reported outcomes, and the lack of homogeneity in quantitative outcomes, the systematic review without meta-analysis (SWIM) guidelines were used for reporting [25].

## Results

### Study selection and individual study characteristics

**Fig. 1:**
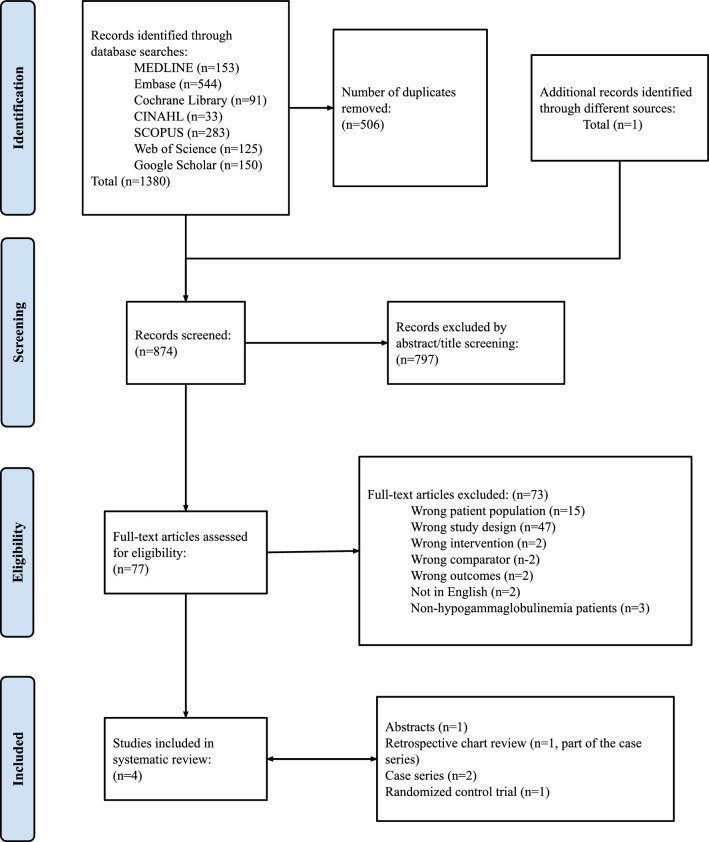
PRISMA flow diagram outlining the systematic selection of the studies starting from the general search of immunoglobulin treatment and COPD to the selection of the four final studies that were included in the review for analysis

The PRISMA flow diagram summarizes the results of the study selection process (Fig. 1). A total of 1381 references were identified after a comprehensive literature search. Removing the duplicates resulted in 874 unique abstracts being screened for relevance, and 77 articles were assessed for full-text eligibility. We excluded 73 records based on the following exclusion criteria: wrong patient population (n = 15), wrong study design (n = 47), wrong intervention (n = 2), wrong comparator (n = 2), wrong outcomes (n = 2), no translation of the abstract or article in English (n = 2), and studies not reporting that the patients had hypogammaglobulinemia (n = 3).

Studies with COPD patients or patients with hypogammaglobulinemia as a subpopulation of a larger target population that did not report individual outcomes specific to the subpopulation above were excluded. Authors of these studies were contacted but did not respond. Initially, ten studies and abstracts were included. Three studies were excluded as the patients were reported to have specific antibody deficiency (SAD), or insufficient data was provided for whether the patients had hypogammaglobulinemia before starting IgG replacement therapy [26–28]. One study reported on outcomes of chronic pulmonary disease which presents itself phenotypically different from COPD due to causal factors such as common variable immunodeficiency (CVID) [29]. Another two studies were conducted with a subpopulation of patients with COPD and hypogammaglobulinemia, but the specific outcomes were not reported [30, 31]. Authors for these studies were contacted to obtain raw data, but the data was not provided.

Four studies were included after full text review. Two studies were retrospective case series, two were retrospective chart reviews (one of which was available in conference abstract form only) and one was a RCT [32–35]. The studies were published between 2010 and 2021, two studies published in Canada [33, 35], and two in the United States [32, 34]. The patient population all had COPD and had hypogammaglobulinemia with a significant proportion of patients from two studies had CVID [32, 34]. The excluded studies and the reasons for their exclusion are available upon request. A detailed description of the included studies is provided in Table 2, and the specific outcomes measured for each study are found in Table 3.

**Table 2:** Study characteristics of all studies included in the systematic review^†^

Study	Study design	Demographic characteristics	Intervention	Baseline clinical characteristics		
				Measured baseline characteristics	Treatment group	Control group
Baleeiro and Mull [32]^31^	Study Design: Case series (Abstract only) Enrollment Dates: 2007–2010 Location: United States Funding: NR Possible Conflicts of Interest: NR	*N* of COPD patients = 17 patients Method of Recruitment: Clinic patients Sex: NR Mean Age (SD): NR Smoking History: Most cases tobacco related Comorbidities: Bronchiecstasis, CVID	**Treatment Group:** Treatment Administration: IVIG or SCIG Dosage: NR Duration of intervention: NR	PFT, Ig, and other parameters of interest were not reported	PFT Results: NR Ig Levels: NR Other Parameters: NR	**No control group**
Cowan et al. [33]^29^	Study Design: Retrospective Case series Enrollment Dates: 2008 – 2014 Location: Canada Funding: None Possible Conflicts of Interest: None	*N* of COPD patients = **14 patients (out of 33 subjects with COPD that had Ig treatment).** *(Among exclusions: 11 not firm diagnosis of COPD, 5 had incomplete records, 3 excluded because* < *3 months treatment due to intolerance of therapy)* Method of Recruitment: Other: Review of medical records Sex: 6 (43%) males, 8 (57%) females Mean Age (SD): 67.1 ± 12.1 Smoking History: NR Comorbidities: 8 cases bronchiectasis 7 cases with asthma 5 cases obese (BMI > 30) 2 CAD 1 diabetes 1 monoclonal gammopathy **No control group**	Treatment Administration: 6 on IVIG, 7 on SCIG, 1 switched from IVIG to SCIG due to intolerance Dosage: 0.5 ± 0.3 g/kg/month Duration of intervention: 363.1 ± 6.9 days	FEV1, L/sec: FEV1, %: FEV1/FVC, %: N patients with severe COPD Mean serum IgG, g/L Mean serum IgG < 7 g/L Mean serum IgG < 5 g/L Other Parameters: Mean rate of moderate/severe AECOPD per year: Mean rate of moderate AECOPD per year: Mean rate of severe AECOPD per year:	PFT Results: 1.2 ± 0.8 46.3 ± 18.6 43.4 ± 15.3 8 Ig Levels: 6.2 ± 2.2 9 (hypogammaglobulinemia) 5 out of the 9 (significant hypogammaglobulinemia Other Parameters: 4.65 3.8 (53 total exacerbations) 0.86 (12 total hospitalizations) 12 hospital admissions occurred in seven of fourteen cases	**No control group**
Cowan et al. [35]^32^	Study Design: RCT Enrollment Dates: September 2016 (inpatients) and March 2018 (outpatients) -Nov 2018 Location: Canada Funding: The Ottawa Hospital Academic Medical Organization Innovative Fund, CSL Behring, and Grifols, with JC receiving grants and/or personal fees from the mentioned organizations – funders not involved in study design, conduct, analysis or interpretation	(444 assessed—failed inclusion criteria (159), refused research (114), failed exclusion criteria (75) failed both inclusion and exclusion criteria (21) died while admitted (5) **Treatment Group:** *N* of COPD patients = 35 Method of Recruitment: Inpatients and clinic patients Sex: 20 males:15 females Mean Age (SD): 66.7 ± 7.4 years Smoking History: Pack years = 56.8 ± 27.5 Comorbidities: - Hypertension = 57% - Coronary artery disease = 26% - Congestive heart failure = 11% - Cardiomyopathy = 8% **Control Group:** *N* of COPD patients = 35 Method of Recruitment: Inpatients and clinic patients Sex: 13 male:22 female Mean Age (SD): 68.7 ± 8.7 years Smoking History: Pack years = 51.3 ± 29.8 Comorbidities:—Hypertension = 57% - Coronary artery disease = 43% - Congestive heart failure = 3% - Cardiomyopathy = 8%	**Treatment Group:** Received IVIG (10% solution) q4 ± 1 weeks for 48 weeks Dose: 0.8 g/kg for hospitalized patients with hypogammaglobulinemia, and 0.5 g/kg for all others **Control Group:** Received IV Normal Saline (NS) q4 ± 1 weeks for 48 weeks Dose: same volume with what they would need if received IVIG In both cases: 1st dose in hospital for inpatients—all other doses at clinical investigation unit	FEV1, L (%): FEV1/FVC, %: IgG, g/L: IgM, g/L: IgA, g/L: IgE, mg/L Other Parameters: Total number of AECOPD, number/year: Moderate AECOPD (managed as outpatient), number/year: Moderate AECOPD (ED visit), number/year: Severe AECOPD (hospitalization), number/year:	PFT Results: 0.90 ± 0.39(34 ± 13) 42 ± 12 Ig Levels: 8.94 ± 2.83 0.85 ± 0.76 2.69 ± 1.15 0.96 ± 1.52 Other Parameters: 2.7 ± 1.3 1.37 ± 1.44 0.34 ± 0.68 1.1 ± 0.8	PFT Results: 0.87 ± 0.4(35 ± 14) 43 ± 16 Ig Levels: 7.34 ± 1.64 0.79 ± 0.56 2.17 ± 1.27 0.35 ± 0.57 Other Parameters: 2.5 ± 1.3 0.86 ± 1.04 0.26 ± 0.56 1.4 ± 0.81
McCullagh et al. [34]^30^	Study Design: Case series Enrollment Dates: January 2012 (January 2010 for the retrospective chart review patients) -Dec 2014 Location: United States Funding: None Possible Conflicts of Interest: None	*N* of COPD patients = **7 patients are included in the case series and retrospective chart review** Therapeutic effect: 7 on IVIG (although the immune diagnosis was associated with the chosen therapy) (3 out of 7 of these patients were also on prophylactic antibiotics **Treatment Group:** *N* of COPD patients = IVIG (7) Method of Recruitment: Other: Clinic patients and Retrospective chart review Sex: NR Mean Age (SD): NR Smoking History: pack years, median (Range): 54 (30–75) Comorbidities: Total - All with CVID	*Treatment Group:* Treatment Administration: IVIG OR SCIG for patients with CVID Dosage: IVIG was administered every 3 or 4 weeks, and SCIG was administered every 1 or 2 weeks The total dose of IVIG or SCIG ranged from 300 to 600 mg/KG/4-week period Duration of intervention: - 1 year	FVC (Range) FEV1 (Range) FEV1/FVC (Range) IgA, g/L (Range) IgG, g/L (Range) IgM, g/L (Range) Other Parameters:	PFT Results: 64.3 (59–68) (n = 3) 41.8 (28–60) (n = 4) 42.3 (30–62) (n = 4) Ig Levels: 0.793 (0.04–2.26) 3.556 (1.11–7.64) 0.359 (0.05–0.63) Other Parameters: NR	**No control group**

AECOPD; acute exacerbations of COPD, NR; not reported

**Table 3:** Specific outcome parameters of studies

Study	Treatment and control population outcomes
Baleeiro and Mull [32]^31^ Study design: Case series	All have reported an improvement in symptoms and a decrease in sputum production and frequency of exacerbations No other parameters measured
Cowan et al. [33]^29^ Study Design: Retrospective Case series	-Mean rate of moderate/severe AECOPD 0.64 (81% decrease, p = 0.0001) - Mean rate of moderate AECOPD 0.57 (decrease 66.7%, p = 0.001) - Mean rate of severe AECOPD 0.07 (decrease 46.4%, p = 0.016) - 1 hospitalization—reduction of mild exacerbations also was present, but numbers not reported due to the subjective nature of the data **Changes in AECOPD events/patient-year stratified by GOLD**[5]** COPD stage and baseline IgG** Moderate COPD (n = 7) pre: 5.14 ± 2.85, post: 0.86 ± 1.13 Severe/very severe COPD pre: 4.14 ± 2.95, post: 0.43 ± 0.5 No bronchiectasis (n = 6) pre: 4.00 ± 2.8, post: 0.83 ± 1.07 With bronchiectasis pre: 5.13 ± 2.93, post: 0.50 ± 0.71 IgG ≥ 5.9 g/L (n = 7) pre: 4.29 ± 3.33, post: 0.29 ± 0.45 IgG < 5.9 g/L pre: 5.00 ± 2.45, post: 1.00 ± 1.07 **Adverse Events** Among n = 14, 1 had transfusion reaction to IVIG and 13 did not report any adverse reaction
Cowan et al. [35]^32^ Study Design: RCT	Study treatment adherence: 68.8 ± 5.7% (median: 85%) 18 patients (51.4%) adhered to 80% of their allocated treatment Study control adherence: 59.4 ± 6.3% (median: 57%) 16 patients (45.7%) adhered to 80% of their allocated treatment Study retention rate (treatment): 97.1% (n = 34). 1 withdrew consent. 3 died during the study period Study retention rate (control): 91.4% (n = 32). 2 withdrew consent. 4 died during the study period **Intention-To-Treat (ITT):** Total number of AECOPD (number of patients with event): 56 (25) treated population & 48 (21) control population Ratio rate (95% confidence intervals): 0.91(0.59–1.41) AECOPD requiring hospitalization (n of patients with events): 16 (13) treated population & 20 (2) control population Ratio rate (95% confidence intervals) 0.78 (0.37–1.65) AECOPD requiring ED visits (n of patients with event): 12 (9) treated population & 2 (2) control population Ratio rate (95% confidence intervals) 3.16 (0.64–15.60) AECOPD managed as outpatients (n of patients with event): 28 (15) treated population & 26 (12) control population Ratio rate (95% confidence intervals) 0.69 (0.37–1.30) **Per Protocol (PP):** Numbers of AECOPD (number of patients with event): 24 (12) treated population & 4 (3) control population Ratio rate (95% confidence intervals): 0.98 (0.50–1.93) AECOPD requiring hospitalization (n of patients with events): 4 (4) treated population & 4 (3) control population Ratio rate (95% confidence intervals): 0.43 (0.07–2.49) AECOPD requiring ED visits (n of patients with event): 5 (4) treated population & 1 (1) control population Ratio rate (95% confidence intervals): 2.52 (0.23–27.7) AECOPD managed as outpatients (n of patients with event): 15 (9) treated population & 19 (9) control population Ratio rate (95% confidence intervals): 0.88 (0.39–1.97) **Adverse events (AE):** 137 AE in 33 treated patients; Median (IQR): 2 (1–5) 126 AE in 27 control patients; Median (IQR): 3 (1–5) (p = 0.55 compared to control) **Tolerability:** A higher number of patients continued to receive study treatment at week 48 in the IVIG group (58%) vs the control group (46%) Treatment adherence for the treatment group was slightly better than the control group; however, no statistical analysis was done
McCullagh et al. ^*30*^ Study Design: Case series	*Treatment Group:* PFT Results: FVC, % (Range): 75.3 (57–93) after IVIG treatment (n = 4) FEV1, % (Range): 51.4 (34–70) after IVIG treatment (n = 5) FEV1/FVC, % (Range): 50.4 (27–69) after IVIG treatment (n = 5) **Outcome Parameters after Treatment:** Number of exacerbations per year (Range): 3.7 (2–6) (n = 7) before IVIG treatment and 1 (0–4) (n = 6) after IVIG treatment Hospitalizations for AECOPD (Range): 2.3 (1–5) (n = 7) before IVIG treatment and 0.83 (0–4) (n = 6) after IVIG treatment ICU admissions for AECOPD: 0.1 (0–1) (n = 7) before IVIG treatment and 0 (n = 6) after IVIG treatment Average number courses of prednisone per year (Range): 6.9 (0–12) (n = 7) before IVIG treatment and 2.5 (0–12) (n = 6) after IVIG treatment Average annual number courses of antibiotics (Range): 6.7 (3–12) (n = 7) before IVIG treatment and 8.67 (2–12) (n = 6) after IVIG treatment Oxygen use (Range): 0.6 (0–1) (n = 7) before IVIG treatment and 0.6 (0–1) (n = 7) after IVIG treatment

AECOPD; acute exacerbation of COPD, NR; not reported

In three of the four included studies, baseline clinical characteristics consisting of PFT results, Ig levels, and other specific AECOPD parameters (hospitalizations for AECOPD events, ED visits, total exacerbations) showed poor lung health, as expected. The main outcomes of interest varied extensively between studies as recorded in Table 2. Baleeiro and Mull qualitatively explained an improvement in symptoms and decrease in frequency of exacerbations [32]. The three other studies quantified changes in AECOPD frequency associated with IRT by assessing hospitalizations, ED visits, and/or systemic glucocorticosteroid prescriptions [33–35].

### Risk of bias results

**Fig. 2:**
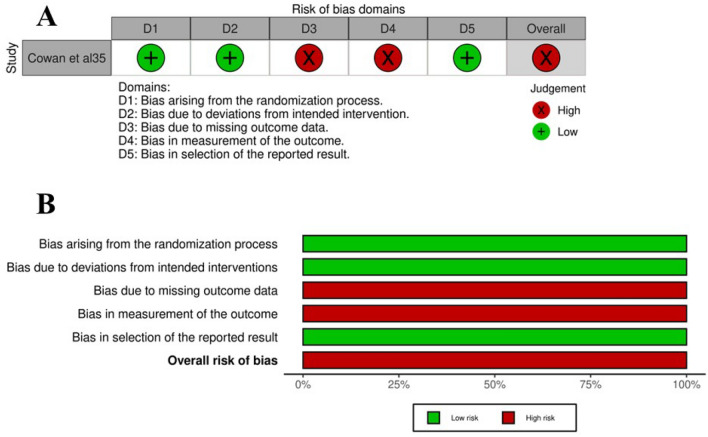
**A** Traffic light plot and **B** summary plot that depicts the risk of bias assessments for each domain using the Cochrane Risk of Bias assessment tool for randomized controlled trials

The risk of bias for the RCT was high and it was deemed as poor quality using the Cochrane risk of bias assessment tool due to its poor treatment adherence [23, 35]. One study, which was available only in abstract form, also had a high risk of bias as assessed using the NHLBI assessment tool as the study design and outcomes data provided were limited [24, 32]. Two studies had a low risk of bias and were assessed as good quality studies using the NHLBI tool [24, 33, 34]. Figure 2a, b and Table 4 outline the assessment of risk of bias for each study in more depth.

**Table 4:** Risk of bias assessments for each criterion using the NHLBI tool for case series studies

Study	Was the study question clearly stated?	Was the study population clearly described?	Were the cases consecutive?	Were the subjects comparable?	Was the intervention clearly described?	Were the outcomes clearly defined, valid, reliable, and applied consistently?	Was the length of the follow-up adequate?	Were the statistical methods well described?	Were the results well described?	Quality of Study
Baleeiro and Mull^32^	Yes	No	NR	No	No	No	NR	No	No	Poor
Cowan et al^33^	Yes	Yes	NR	Yes	Yes	Yes	Yes	Yes	Yes	Good
McCullagh et al^34^	Yes	Yes	NR	No	Yes	Yes	Yes	Yes	Yes	Good

NR, not reported

## Discussion

Using a rigorous systematic review process, we identified four studies that evaluated COPD outcomes after IGT. The main objective was to determine if COPD patients with low IgG serum concentration would benefit from IGT to prevent AECOPD.

One pilot placebo controlled RCT designed to address this question was identified [35]. Seventy patients were randomized to receive IVIG or placebo (1:1) for 48 weeks. The patients had very severe baseline airflow limitation with an average FEV1 < 1L. The authors found no difference in the frequency of all AECOPD or AECOPD needing hospitalization between the placebo and treatment groups. There was a trend of improvement in the time-to-first AECOPD event. No difference in pulmonary function measures was seen between the treatment and placebo groups.

These findings controverted prior observational studies that found a significant reduction in the frequency of moderate and severe AECOPD [32–34]. However, these observational studies were limited by their retrospective design, small sample sizes, and possible referral bias as they were conducted in conjunction with immunodeficiency clinics. The observational studies also included a higher proportion of patients with moderate *versus* severe airflow obstruction compared to the RCT, two included patients with comorbid post-infectious bronchiectasis [32, 33] (a possible independent marker of an antibody deficiency syndrome), and one only included patients with demonstrable impairment in specific antibody production [34].

There was significant heterogeneity in reported outcomes across the studies. This issue is well known in the COPD research community and has spurred movements such as the DisEntangling Chronic Obstructive Pulmonary Disease Exacerbations clinical trials NETwork (DECODE-NET) [36], which has proposed a core outcome set to improve standardization [37]. Hospitalizations and ED visits were more consistently reported in the studies identified, but these may also not be the most informative metrics. A systematic review found that time-to-event may be a more suitable outcome parameter than length or frequency of hospitalization stays due to extraneous factors like social circumstances or comorbidities [38]. Additionally, patient reported outcomes reflecting symptom burden and quality of life, should also be included [37].

Given the paucity of data in this area, further studies are greatly needed. An important finding of the RCT was that treatment adherence for both the IVIG and control groups was low—68.8 ± 5.7% and 59.4 ± 6.3%, respectively [35]. This observation indicates that additional trials with this design may be challenging to conduct. Future studies should thus consider using SCIG instead of IVIG as it is better tolerated, does not require venous access, and is more cost effective [21].

Improved patient selection may also be critical for the successful use of IRT in patients with COPD and reduced serum IgG. First, given the issues with treatment adherence and cost, IRT studies should particularly target COPD patients who have recurrent AECOPD despite maximal therapy including combined glucocorticosteroids long-acting muscarinic antagonists, and long-acting beta-agonist inhaler [39]. Second, biomarkers that predict the type of AECOPD for which an individual is at risk may be important. For example, patients with type 2 airway inflammation evidenced by blood eosinophils > 300/µL may represent a distinct pathophysiological subgroup [6] that is better managed with anti-type 2 biologicals such as dupilumab [40]. COPD patients with recurrent infective exacerbations due to *Haemophilus influenzae* and *Moraxella catarrhalis* may derive benefit from chronic azithromycin [41], and a trial of azithromycin may be warranted prior to trying IRT. Third, assessment of specific antibody production by measuring vaccine response to peptide and polysaccharide vaccines may better identify COPD patients with defective humoral immunity. Memory B cell enumeration by flow cytometry may also be useful, particularly to differentiate between low IgG from systemic glucocorticosteroid use and impaired IgG production [42]. However, these measures will increase the cost and complexity of patient evaluation. Fourth, it is possible that patients with moderate rather than severe airflow obstruction would benefit most from IRT. Structural changes of the lungs, including advanced airway remodelling and emphysematous destruction of the pulmonary parenchyma rather than defective adaptive immunity, may be the main driver of increased infection susceptibility in patients with advanced COPD.

## Conclusion

There is currently insufficient evidence to support the routine use of IGT in COPD patients with low IgG to prevent acute exacerbations. The quality of currently available data is low due to poor IGT adherence, small sample sizes, heterogeneity of the populations studied, and incomplete outcome reporting. Additional well-designed, prospective studies are needed to address this important clinical question. Future studies should select COPD patients with recurrent exacerbations despite optimized therapy and use SCIG instead of IVIG given its better tolerability.

### Supplementary Information

**Additional file 1.** PRISMA 2020 Checklist.
